# Bridging the Gap: The Role of Parent–Teacher Perception in Child Developmental Outcomes

**DOI:** 10.3390/children12091260

**Published:** 2025-09-19

**Authors:** McKayla Jensen, Mikaela J. Dufur, Jonathan A. Jarvis, Shana L. Pribesh

**Affiliations:** 1 Department of Sociology, Brigham Young University, Provo, UT 84602, USA; mckaylajensen16@gmail.com (M.J.); jonathan_jarvis@byu.edu (J.A.J.); 2 Department of STEM Education and Professional Studies, Old Dominion University, Norfolk, VA 23529, USA; spribesh@odu.edu

**Keywords:** social development, cognitive development, behavior, early childhood education, childcare, parent–teacher cooperation

## Abstract

**Highlights:**

**What are the main findings?**

- Parent and teacher differences in perceptions of young children’s behavior regarding desire for knowledge, confidence, talkativeness, good-naturedness, and understanding are negatively associated with at least one key developmental metric (social, concentrative, general knowledgeability, linguistic, and mathematical).
- Parent and teacher differences in perceptions of a child’s behavior regarding neatness are positively associated with all five developmental metrics.

**What is the implication of the main finding?**

- Differences in how parents and teachers perceive child behavior are significantly associated with the development of children’s social and cognitive abilities compared to their peers. Aligning parent and teacher perceptions of children may help children reach developmental milestones in a timely manner.

**Abstract:**

Background/Objectives: Time spent with parents and educators encompasses a large portion of a child’s waking hours, with the home and early childhood education and care serving as two of the first settings in which children develop social and cognitive abilities. While previous studies have used social and cognitive tests to examine antecedents of child behavior, we extend such studies to take into account the congruence and incongruence of parents’ and teachers’ views on those antecedents. We examine the importance of parent-teacher alignment on the perceptions of a child’s personality and abilities in early development. Methods: Parents and teachers of 2968 German Kindergarten-aged (4–5 years old) children were surveyed using the National Educational Panel Study (NEPS). Parents and teachers independently rated 10 child behavioral traits, with higher scores indicating more prosocial behavior. Educators also rated children on five developmental abilities (social abilities, ability to concentrate, language abilities, general knowledgeability, and mathematical reasoning) compared to the student’s peers. While previous work has often examined how parental investments in children or teachers’ views of children might be related to development, we provide a new take by examining parents and teachers in conjunction with each other. Research that has looked at both parents and teachers has tended to examine alignment, or lack thereof, on child behaviors and personality traits. We analyzed child developmental abilities using OLS regression models, measures of parent–teacher divergences in ratings of child behavior, and demographic controls. Results: Greater differences in parent and teacher perceptions of desire for knowledge were negatively associated with all five developmental abilities. Differences in parent and teacher perceptions on talkativeness, confidence, good-naturedness, and understanding were negatively associated with at least one developmental outcome. By contrast, differences in perceptions of children’s neatness were positively associated with all five developmental abilities. Conclusions: Using both parent and teacher perceptions of child behaviors and abilities is a unique approach to understanding the relevance of parent and educator perceptions to a child’s development. Our findings indicate the need for collaboration across young children’s home and school or care settings. Establishing congruence in perceptions and the kinds of relationships that can lead to such congruence can help children with behavioral issues receive support in both home and educational settings and encourage mutual respect and partnership between parents and educators.

## 1. Introduction

Very young children require nearly constant supervision, and the adults in their lives are the most important agents of children’s early social and cognitive development. For most children, these adults include first their parents, but then the scope of adult influence broadens to include non-familial caregivers and educators. As a result, the home and the preschool become two of the most pivotal arenas for adult influence and intervention for child development. As young children learn and grow, they are exposed to new ideas, skills, and personalities, and their ability to grasp new concepts increases in complexity [1]. While positive gains in development are commonplace [2], progress can be helped by a myriad of supportive practices, both at home and in preschool. For example, toddlers taught about the concept of empathy foster more prosocial interactions with peers [3]. Children coached in delayed gratification and “effortful control” learn to accept rules and regulate emotions [4]. Integrated opportunities to create stories and imaginary games with siblings or peers are practices in managing social relationships and interactions, which serve to increase a child’s cooperative nature [1,5]. Equipping children with tools to engage in new social situations can help reduce behavioral issues and support social and emotional skill building [6]. Children who are provided ample resources to nurture new relationships both at home and beyond the home often possess more prosocial attributes. However, children who are not given these tools or who might experience deficits or disagreement at or between home and non-familial care could experience slower development. In this paper, we investigate how misalignment across adult actors’ perceptions of the child in these two important settings of home and preschool might be related to early childhood development. We examine children attending Kindergarten in Germany, where the Kindergarten experience is characterized both as caretaking of the child outside the home and, as children approach school age, preparation for transition to formal schooling.

### 1.1. Developmental Differences and Influences

Understanding how well a child performs regarding certain metrics of social or cognitive ability can be pivotal in establishing plans to magnify strengths and bolster weaknesses. Understanding a child’s background also highlights potential traits or abilities that may need more support. Some developmental areas, such as reading and numbers, are predicated upon language and spatial comprehension [5]. Academic and social success often differ by social group [2,7]. Girls may present more outward displays of sociability and competence, whereas boys are more often cited as having behavioral issues [2]. Children from higher socioeconomic households tend to allocate more time and resources into ensuring social and academic success via extracurricular activities, and what is sometimes referred to as “concerted cultivation” [8,9]. Some research suggests children who have a larger number of siblings receive lower parental investments, leading to deficits in early academic success [10]. Additionally, children whose parents and teachers are aligned in educational expectations can benefit from stronger, consistent support from both caregivers [11,12]. Understanding traits, both intrinsic and extrinsic, that may affect a child’s development is key to enabling children to learn and grow at a steady pace.

Caretakers play a major role in facilitating successful social and cognitive gains. Balancing flexibility and child independence can be an intricate process for parents and educators alike [13]. Children whose caretakers invest time and attention to help them develop abilities often outshine children whose development is not as carefully monitored or guided [5,13,14]. Supporting children’s emotional development and self-concept is also key to building trusting relationships where active learning and observation by the child can take place [3,15]. Intention and consistency can help caretakers to maximize a child’s social and cognitive development from an early age.

### 1.2. Establishment of Behavior: Issues and Supportive Strategies

As a child’s abilities develop, their behaviors and tendencies also become more fixed. When behaviors are problematic, there is a greater likelihood that the issues are present across multiple environments, such as homes and non-familial care or school [16]. Variations in behavior across different settings, however, may result in differing perspectives among parents and educators [17]. Conversely, parents and educators can report congruent assessments of behavior, whether positive or negative, that can serve as valuable indicators of the consistency and establishment of a child’s personality and conduct [16]. Such congruence can help adults across settings take actions to modify or to reinforce child behavior, or to intervene when needed in promoting child development.

As young children undergo the process of learning and interacting with others, their academic success, social prowess, and behavioral patterns develop under the attentive guidance of both parents and educators. This developmental journey is influenced by a myriad of factors, one of which is the quality of communication and mutual respect between a student’s teachers and parents [18]. Behavioral challenges are not uncommon among young children, and persistent problematic behaviors across various settings may signify underlying issues [19]. At school in particular, when a child repeatedly engages in negative behavior, it can impact the quality of their relationship with their educator, leading to adverse shifts in how the educator views the child [16,20]. When parents and teachers fail to address antisocial behavior, the child may act out more often and more aggressively [13]. When communication between parents and teachers is not regular or intentional, supporting children in developing prosocial traits may be less efficient than if strategies for growth are premeditated and practiced by both parties.

Positive examples of cooperation between parents and teachers often reflect how a child interacts with authority figures in their lives as well. At home, children benefit from warm parental guidance, which fosters prosocial behavior and nurtures emotional regulation skills through validation and encouragement of positive actions [21]. Similarly, educators and caretakers employ social and academic resources to encourage prosocial behavior and academic growth in the children they teach. Stories and games that promote empathy and mindfulness are powerful mechanisms to guide children’s behavior [3]. With support from both parents and teachers, children are exposed to a wealth of opportunities for cultivating prosocial conduct and cognitive abilities.

### 1.3. Parental Direct and Indirect Influences on Early Development

As the earliest socializing agents in a child’s life, parents can be deliberate in helping establish a child’s first proclivities to pro- or antisocial development and cognitive functioning. Different parenting styles can be more or less conducive to a child’s progress in establishing new abilities. Children whose parents address conflict with words rather than punishment can learn more effective self-regulation skills to mediate their own future disputes [2]. Taking more time to talk through issues with children also helps increase their vocabulary, which can lead to more significant gains in the classroom when it comes to socializing and building linguistic abilities [22]. Unconditional warmth from parents also establishes homes as safe spaces for children to practice cooperation and reciprocate positive acts [21]. Children whose parents are more authoritative, with high levels of warmth, support, and consistent expectations, are often the most deliberately prepared for socializing and developing outside the home [14]. Authoritative parents are more likely to have attained higher education and place an emphasis on learning for their children [22]. Permissive parenting, on the other hand, can yield mixed results. Some permissive parents are loving and seek out the best for their children, but others promote freedom and fewer boundaries, leaving children to cultivate social and cognitive abilities largely unsupervised [13]. When parents seek to implement the best practices for supporting their child’s development, it can enable the child to accelerate at a faster rate than their peers.

While most parents are actively involved in their children’s development, it is also true that parents are likely sensitive to others’ perceptions of their children, and a number of personal characteristics might influence how parents perceive their children’s growth. Parental self-efficacy, for example, may not only affect a parent’s ability to regulate their own emotions and help their child do the same, but also a parent’s ability to accurately assess their child’s behavior and growth [23]. Along the same lines, parental depression, particularly maternal depression, can lead to parents having less energy to spend on cultivating a child’s abilities, as well as to possible faulty perceptions of both their own parenting performance and their child’s development [24]. Social class may also affect both development and parents’ perceptions about their children. Parents of lower SES households may lack the time or resources to consciously engage with their children in ways that encourage their active learning in the same way as parents in higher SES households with more time can do [9]. Financial stress and struggles, the lack of energy or time, and other issues may cause more psychological distress for parents trying to manage numerous responsibilities to keep their household afloat, which can make it much more difficult to manage a child’s difficult disposition [22]. There is also evidence that social class affects what development milestones and opportunities parents value most [25]. As the earliest catalysts for social and cognitive development, parents bear the weight of driving child development from infancy into toddlerhood. While this can manifest as children being exceptionally prepared to thrive in social circles and develop cognitive competence later on, other children may be markedly behind due to a lack of resources or support from their families or because their parents’ perceptions of their developmental needs are misaligned with their actual needs.

### 1.4. Educator Direct and Indirect Influences on Early Development

As young children approach the later stages of toddlerhood, they often begin interacting in more settings outside the home, whether it be in educational or other contexts. As children enter new environments, spending time with educators and peers, they are apt to develop new social and cognitive abilities at an even greater rate than before, when they socialized primarily at home. Children in early learning facilities often form bonds with their teachers, who seek to cultivate their linguistic, numerical, social, and emotional abilities directly. Preschools that offer training to keep educators up to date on their own social and emotional management skills, as well as strategies to mitigate behavioral issues by students, can improve child–teacher relationships [6]. The specific language choices educators use when interacting with young children are also paramount to maintaining attention and effectively refining a child’s burgeoning developmental abilities [5]. A teacher’s training and strategies often give them a broad perspective on a child’s cognitive ability development, which makes them effective gauges for a child’s development [26]. Their training and experience might also lead them to make more accurate assessments of children’s growth than parents.

Similar to parents, however, educators are subject to having unconscious biases and indirect effects on how well they can support child development. Educators may show gendered biases or expectations in preschool settings, as some toys may be directed to one gender over the other [2]. Additionally, teachers often report closer relationships with female students, as boys are more likely to exhibit problematic behavior [20,27]. Teachers often score students who are prone to aggression or other behavioral issues lower on ability assessments, perhaps because teachers in such situations find themselves having to focus more time on solving conflicts instead of teaching [15,20]. When educators struggle with either their own emotional regulation or in teaching children better regulation skills, this can negatively affect child development [24]. On a broader scale, children’s social abilities and adjustment to preschool are affected by the quality of the daycare facility and the degree of support in the classroom [28]. Teachers who view their students’ behavior as more problematic, or who experience disagreement with parents, might struggle to provide as much classroom support for certain students. As children spend more time outside the home, the quality of new environments can serve to accelerate or decelerate development, but this may be dependent on the ability of teachers to align assessment and perceptions with parents.

### 1.5. Parent and Teacher Perceptions Intertwined

Although parents and educators largely interact with a child in separate spheres, their relationships with the child can be interlinked, and both parties have unique insights into how a child is developing. When parents and teachers have a higher-quality relationship, it often yields closer collaboration in cultivating a child’s abilities [18]. Children in this situation experience consistent encouragement and support in both settings, leading to more positive gains in social and cognitive development. For teachers, a shared, positive view of their relationship with a student’s parent often results in favorable reports of a child’s behavior and capabilities, extending beyond mere academic achievements [26,29]. Conversely, negative perceptions of the parent–teacher relationship correlate with teachers’ reports of the student’s behavior, and those reports are more likely to highlight problems. This may suggest that any lack of congruence between educator and parent assessment will be more influential on the child’s development outside the home than in the home. This may be especially true for children from more resource-rich class backgrounds, who might view educators or caretakers as employees rather than colleagues [25]. As parents and teachers both play major roles in a child’s early development, deliberate teamwork can establish the optimal, consistent conditions through which to bolster development. It is less clear if a lack of that teamwork has a negative effect on child development.

### 1.6. The Present Study

In this study, we aim to develop a greater understanding of how disparities in parents’ and teachers’ views of child behavior affect social and cognitive development. One previous paper using a small sample of troubled children from the United States focused on parent–teacher congruent opinions on the quality of the relationships between parents and teachers and found that lack of alignment with adult actors was associated with social development but not academic development [26]. Our research expands these inquiries to examine how incongruence in opinions about the child can affect development. We also look at a broader set of developmental outcomes and a larger, generalizable sample from a country (Germany) where early childhood education receives strong state support and is, therefore, widely used. To do this, we use responses from both parents and instructors of young children in Germany during their daycare and early schooling years (Kindergarten). We examine both multiple developmental outcomes and multiple opportunities for agreement or disagreement across parents and teachers to explore nuances in how inconsistencies in perceptions or misalignment of opinions on children’s development might be related to that development compared to same-age peers. We expect that in situations where parents and teachers have greater agreement on child behavior and aptitude, children will have better or faster development compared to their peers whose parents and teachers are not in congruence. We also expect that these patterns will hold across different development outcomes and different child characteristics.

## 2. Materials and Methods

### 2.1. Participants

We drew upon data from Starting Cohort Two of the National Educational Panel Study (NEPS), an ongoing initiative conducted by the Research Data Center at the Leibniz Institute for Educational Trajectories (LIfBi) in Bamberg, Germany [30]. NEPS comprises seven starting cohorts encompassing individuals from newborns to adults, with an overall participation of 70,000 individuals. Participants hailing from every state in Germany contributed to the study, providing a comprehensive perspective of German schooling and its outcomes. An additional 50,000 participants, comprising parents and educational professionals, provided deeper insights into the development of each target participant. The study examined the influence of education across different life stages, including pre-education measures such as pregnancy and infant health records, as well as post-education careers, relationships, and lifestyles.

We used the public-use version of Starting Cohort Two (SC 2) of the NEPS data, which commenced in 2010, with about 3000 children and their parents interviewed. At the time of initiation, the children were four years old and attended diverse Kindergarten institutions across Germany. These Kindergartens are similar to daycare facilities in America, though perhaps with more focus on preparation for formal schooling, especially at later ages. For ease of interpretation, we refer throughout the paper to German Kindergarten facilities as preschool. A group of roughly 20–30 children between the ages of three and six is looked after from the morning hours until midday or midafternoon. While no formal learning objectives are established, Kindergarten educators provide children with opportunities to socialize, as well as to gain experience with basic abilities such as learning the alphabet and spelling, counting, and prosocial behavior. In this paper, we refer to the Kindergarten leaders who facilitate the children’s interactions and learning as both “teacher” and “educator.” For the cohort of Kindergarteners in the NEPS, extensive theory-based surveys were administered to the children, their parents, educators, and Kindergarten administrators. Prior to conducting the data collection, a data protection and security officer of LIfBi acquired written informed consent from parents and educational institutions that provided data. Similar to other studies [31,32], our analysis incorporates survey responses from both the target children’s parents and preschool educators, offering insights into the children’s behaviors and social tendencies from both adult perspectives. It is possible that more than one child is enrolled in the same preschool. In such cases, our models might introduce shared error that would violate the assumptions of the regression strategies we use. One strategy to address this issue would be to use clustering methods to adjust for shared error. However, institutional identifying information that would allow for this method is not available in the public-use data. We are reassured that any such shared error is unusual in these data because of the specific sampling strategy. NEPS initially sampled 212 elementary schools and asked those schools for information on the kindergarten/preschools that fed into them. We focused on the children’s experiences in these preschools. The NEPS sampling strategy resulted in 1432 kindergarten/preschools, indicating that shared enrollment in a specific preschool is unlikely. This increases our confidence in the accuracy of the results we report below.

### 2.2. Measures

Social and Cognitive Development: Early child development outside the home is measured using five variables, each considered as separate and distinct from the other. The social ability variable refers to sharing with others and following the rules. The persistence and ability to concentrate variable refers to being able to remain occupied with an activity or task for an extended period of time. The language ability variable refers to vocabulary and sentence construction. The general knowledgeability variable refers to knowing facts about animals, plants, and the environment. Finally, the mathematical ability variable refers to the ability to count and recognize small numbers. Educators were asked to rank a child’s abilities compared to those of their peers in each of these five areas. Responses were given on a scale of 1 (Much poorer than other children of the same age) to 5 (Much better than other children of the same age). These assessments were performed by the Kindergarten leader; these leaders, on average, report children as performing similarly to their peers (see Table 1). We acknowledge the potential weakness of using leader assessments as measures of development while also comparing leader and parent opinions on other items; for example, we might expect leaders’ opinions to have stronger relationships with their own assessments than parents’ opinions do. We note that there are potential drawbacks to solely using teacher ratings for the outcome variables, as previous research has shown how teacher ratings may be affected by outside factors such as mental wellness and parent–teacher relationship [24,29]. Previous research using the NEPS to examine early childhood development used similar outcomes [31,32], and we note that our key independent variables of interest are not the teacher opinions themselves, but rather their divergence from parental opinions. We discuss potential ramifications of using teacher outcome scores in greater detail in the limitations section of our discussion below.

Behavior: To measure child behavior, we used ten traits. Parents and teachers assessed each trait on a ten-point Likert scale, where prosocial behaviors are represented by higher values. Parents and educators were asked to what degree the child exhibits that particular trait. These traits include talkative/quiet, tidy/messy, good-natured/irritable, hungry for knowledge/uninterested, self-confident/insecure, sociable/reserved, focused/easily distracted, obedient/stubborn, understands quickly/needs more time, and fearless/anxious. We then used the parent and educator variables to create a congruence score for each of the ten traits, where the original educator score is subtracted from the original parent score, with the subsequent value given in its absolute form. These congruence variables measure the difference between parent and educator responses for each trait; our purpose here was to examine any dissimilarity between parent and educator, so we did not distinguish which actor assessed the child more positively when incongruence occurs. This allowed for a more comprehensive view of the congruence of parent and educator ratings of a child’s behavior. Future work examining the directionality of parent–teacher ratings could yield further insight into how parent and teacher perceptions matter in different ways. Table 2 demonstrates substantial incongruence between parents and teachers, between 2.5 and 3 points on average on a ten-point scale, depending on the item (see Table 2). Parents and educators assessed children most differently with regard to students being good-natured and fearless and least differently when looking at being sociable or talkative.

Controls: We included a number of household and parental characteristics that have associations with child behavior and development. Household size was measured on a scale of 1 to 6, where each number represents the true number of household members, with the exception of 6, which accounts for households with 6 members or more, with a maximum household size of 11. This was done to reduce the right skew of the household size variable. Monthly household income was measured in categories ranging from 0 to 6, where 0 indicates 0 Euros, 1 indicates 1 to 1000 Euros, 2 indicates 1001 to 2000 Euros, 3 indicates 2001 to 3000 Euros, 4 indicates 3001 to 4000 Euros, 5 indicates 4001 to 5000 Euros, and 6 indicates 5001+ Euros, with a maximum of 75,000 Euros. This variable was aggregated to account for skew. We also asked parents to assess the stability of their household economic situation as an additional measure of social class; this variable was measured on a scale of 1 (Very bad) to 5 (Very good).

In the data used for this study, the child’s primary parent who served as the respondent was typically the mother (91% of total parent respondents) [30]. Primary parent’s employment is a categorical measure including 1 (Full-time employment), 2 (Part-time employment), 3 (Working a side job), or 4 (Being unemployed). Being unemployed includes parents who are stay-at-home caregivers. We note that because of the strong state support for Kindergarten, it is common in Germany for children to attend Kindergarten even when they have a stay-at-home parent. We included two dichotomous variables to tap family structure; the first asks if the primary parent is married to their partner (yes = 1), while the second asks about the primary parent’s partner’s relationship to the target child (biological parent = 1). A final dichotomous variable asks whether the primary parent is a migrant (yes = 0). We also measured maternal age at birth in years. Parent educational attainment was measured in years; we measured this for both the primary parent and the primary parent’s partner. To measure educational attitudes, we used variables that ask parents five statements that capture their views on early education. The statements included “children are put under a lot of pressure to perform in the elementary school,” “at elementary school, children whose performance is weaker receive little support,” “the demands are too high at elementary school,” and “we are losing the fun in learning at elementary school.” Parents responded to these statements on a scale of 1 (Does not apply) to 4 (Does rather apply). We included an additional, similar variable asking parents their belief in the importance that their child is being prepared for school, where responses ranged from 1 (Not at all important) to 4 (Very important). Descriptive statistics for parental variables are in line with population estimates for German parents of young children. We also included measures of the target child’s birth weight (in grams) and sex (male = 1) (see Table 3).

### 2.3. Data Analysis

We used Stata version 18 to perform ordinary least squares (OLS) regression models predicting each of the five developmental abilities separately. Alternative modeling approaches that treated the developmental ability variables as categorical were not better fits to the data and did not produce different patterns of results, so we report the OLS analyses here for ease of interpretation. The original sample included 3344 children. There was a small amount of attrition between waves 1 and 2; in addition, there was a small amount of nonresponse. To address these missing data, we created and ran analyses on 25 imputed datasets using Stata 18’s MICE multiple imputation function. This brought the number of complete cases to 2968; this is the sample we report on below. In our discussion of the results below, we portray the findings as percentages in lieu of regression coefficients. Our figures below portray the percent increase or decrease a child’s teacher’s developmental rating would experience for each point their parent and teacher differed on a particular behavioral rating. This would be a percentage of the 5-point scale teachers used to assess a child’s social and cognitive developmental abilities. The table below also includes standardized coefficients for ease of comparison.

## 3. Results

Table 4 presents regression analyses using each parent–educator incongruence measure to predict developmental abilities (standardized coefficients are in parentheses). We show only the incongruence measures in the table, but each model contains the control measures listed above (results for controls available upon request). Controls behaved as expected in each model; for example, children from wealthier families, with more education, and with older mothers at birth, scored higher on developmental abilities. Girls scored higher on average than boys in social, concentrative, and linguistic skills. Additionally, children from larger households typically scored lower on average.

We describe in detail below the findings for each developmental ability, but overall, parent–teacher incongruence on being neat and being hungry for knowledge exhibited the most consistent associations across developmental abilities, followed by incongruence on understanding things quickly. Interestingly, incongruence between parents and educators on being sociable, focused, obedient, and fearless was not significantly associated with any developmental ability, even though incongruity scores for those items were very similar to those for other items that show more association with developmental abilities.

### 3.1. Social Development

**Figure 1:**
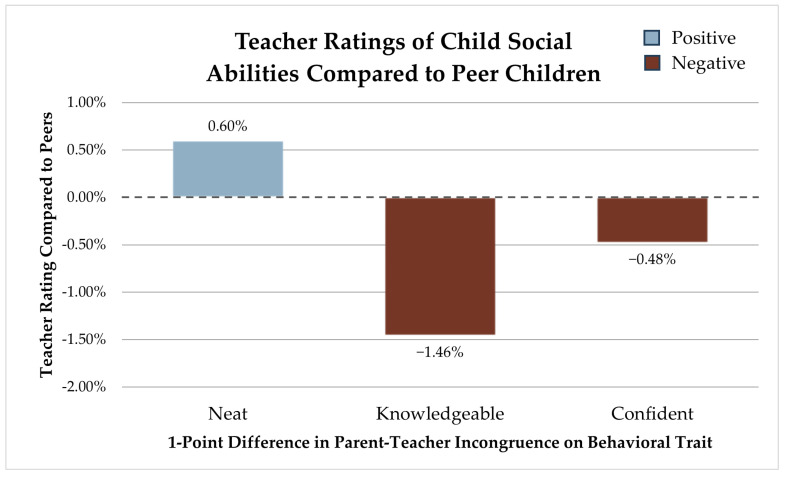
Teacher ratings of child social abilities compared to peer children.

Of the behavior perception differences between parents and teachers, there are three that are significantly associated with social development. For the purpose of making findings more easily interpretable, we converted the regression coefficients for each developmental ability and behavioral perception incongruence to a percentage of how much higher or lower a child’s developmental ability rating would be compared to peers based on the 5-point scale used to measure abilities. In the figure below, behavioral perception incongruencies that yield higher expected developmental ratings are highlighted via blue bars that extend above the zero baseline. Incongruencies that produced lower expected ratings are denoted with red bars that drop below the zero baseline (see Figure 1). Greater variances in neatness ratings yielded better expected social development ratings. For each point of difference in parent–teacher perceptions (max of 10 points), a child is expected to have a 0.60% (SE 0.011) higher social development score compared to their peers. Greater incongruence in knowledge hunger and confidence traits both produced lower expected social ability ratings compared to peers. For each point for which parent and teacher perceptions diverged on desire for knowledge, a child’s social development rating is expected to be 1.46% (SE 0.014) lower. For confidence, a 0.48% (SE 0.011) lower rating is expected for every point parents and teachers differed in their perceptions.

### 3.2. Concentrative Development

**Figure 2:**
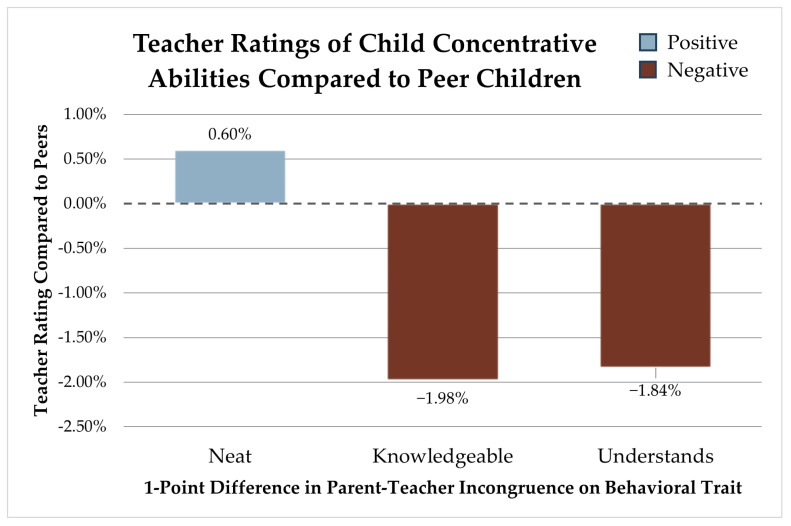
Teacher ratings of the child’s concentrative abilities compared to peer children.

Three behavior perception differences between parents and teachers are significantly associated with concentrative development. Divergence in opinions about neatness, once again, is associated with an increase in a child’s concentrative rating compared to peers by 0.60% (SE 0.010) for each point parents and teachers differ. There are two behavior-perception incongruencies that are negatively associated with concentrative development. Hunger for knowledge yields a 1.98% (SE 0.014) lower concentration rating for each point parents and teachers differ. Additionally, for each point for which parents and teachers differ on how quickly a child understands new concepts, we see a 1.84% (SE 0.011) lower concentrative rating compared to peers (see Figure 2).

### 3.3. Language Development

**Figure 3:**
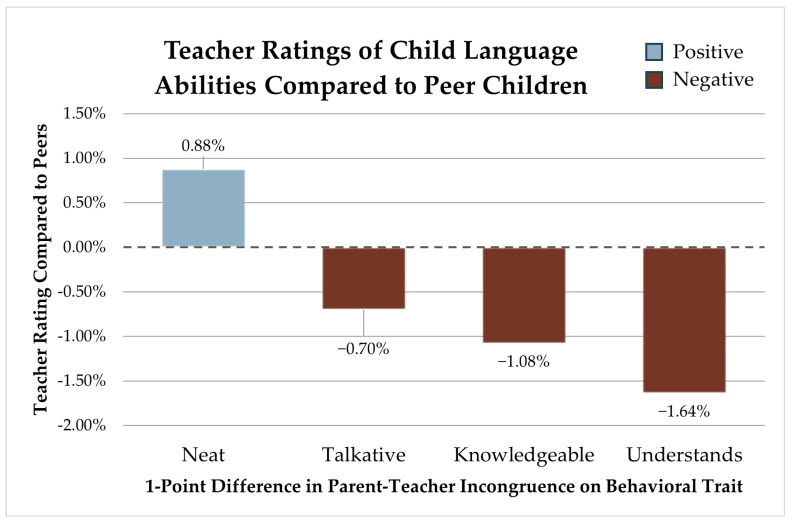
Teacher ratings of the child’s language abilities compared to peer children.

Language development ratings were significantly associated with four behavior perception differences between parents and teachers: neatness, talkativeness, hunger for knowledge, and understanding. For each point in which parents and teachers differ on how neat they perceive a child, we observe a 0.88% (SE 0.012) increase in the child’s language development rating compared to their peers. Each point of variance in parent and teacher talkativeness ratings produces an expected 0.70% (SE 0.013) decrease in a child’s language ability compared to peers. Incongruence in ratings of hunger for knowledge is associated with a 1.08% (SE 0.014) decrease in language development ratings. For each point in which parents and teachers diverge on perceptions of a child’s speed of understanding, we see a 1.64% (SE 0.012) lower language development rating compared to peers (see Figure 3).

### 3.4. General Knowledgeability Development

**Figure 4:**
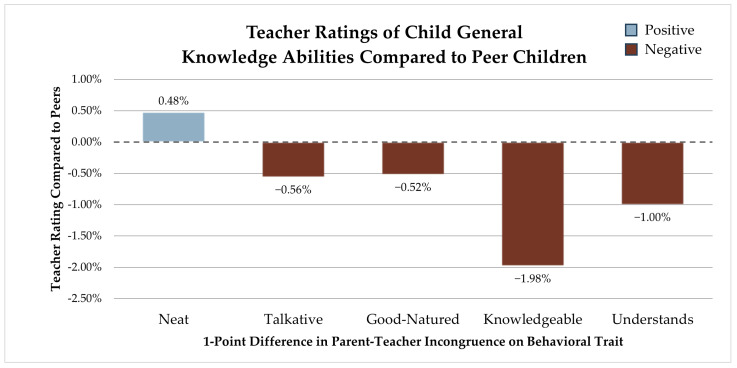
Teacher ratings of the child’s general knowledge abilities compared to peer children.

General knowledgeability developmental ratings compared to peers were significantly associated with five behavior perception incongruences: neatness, talkativeness, good-naturedness, hunger for knowledge, and understanding. For each point in which parents and teachers diverged on how neat they perceived a child to be, we see a 0.48% (SE 0.010) higher knowledgeability rating compared to peers. For talkative perceptions, each point of variance between educators and parents is associated with a 0.56% (SE 0.011) lower knowledgeability development rating. As parents and teachers differ on how good-natured they perceive a child, each point of difference is associated with a 0.52% (SE 0.009) lower knowledgeability rating compared to other children. For each point of incongruence in how much a child hungers for knowledge, a 1.98% (SE 0.012) lower general knowledgeability developmental rating is expected. Differences in how teachers and parents rate how quickly a child gains understanding are associated with a 1.00% (SE 0.010) lower knowledgeability rating each (see Figure 4).

### 3.5. Mathematical Development

**Figure 5:**
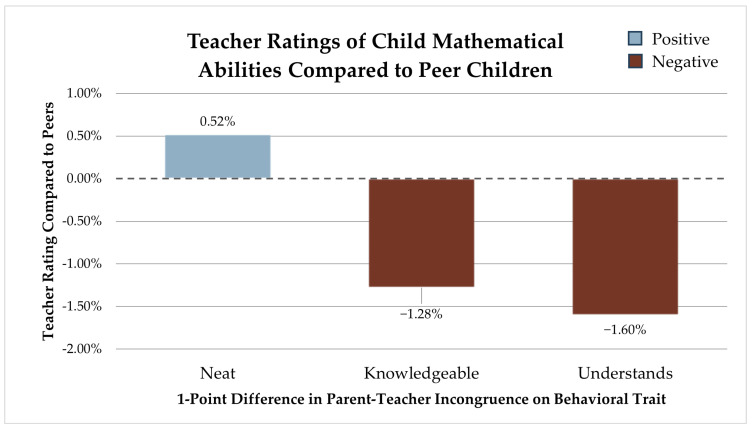
Teacher ratings of the child’s mathematical abilities compared to peer children.

Mathematical developmental ratings compared to peers were significantly associated with three behavior perception differences between teachers and parents: neatness, hunger for knowledge, and understanding. Neatness perception differences between parents and teachers are associated with a 0.52% (SE 0.001) higher mathematical development rating compared to peer children for each point of incongruence. For every point in which parents and teachers diverged on hunger for knowledge, we see a 1.28% (SE 0.011) lower mathematical abilities score. Finally, for each point of variance in how a child’s understanding is perceived, we see a 1.60% (SE 0.011) lower mathematical development rating compared to peers (see Figure 5).

## 4. Discussion

Our study took the perceptions of teachers and parents on ten behavioral traits in Kindergarten-aged children to examine how incongruities between parent and educator perceptions might be related to early childhood development. In some cases, parents may be prone to overestimating their child’s comprehension abilities due to a parent’s difficulty in developing accurate insight into their child’s inner state of mind, or a desire to portray their children favorably when rating behaviors [33]. On the other side, teachers may substantially base their perception ratings of hunger for knowledge and understanding on outside factors such as sociodemographic or gender differences [34]. Either case may lead to instances where a parent’s and a teacher’s behavior perceptions vary more than they otherwise would.

We found mixed but intriguing evidence that such incongruities matter for child development. Discontinuity in perceptions on four of the ten traits was not associated with children doing either better or worse than their peers on the five developmental abilities. However, incongruence on six more traits was associated with worse developmental outcomes in young children. We predicted that parents and teachers being more in synchronization with each other would help promote child development in a number of ways, including modeling positive social relationships with each other and building ties across which parents and teachers execute similar strategies to promote child growth. In the cases of being sociable, focused, obedient, and fearless, this simply was not true. We expected that sociability and focus would be especially likely to be associated with their respective developmental realms, social and concentrative abilities, but this was not the case. Perhaps these dispositions are not as important to parents as they send their children into new social settings for the first time in their young lives, or perhaps they expect that teachers will assess these traits better, resulting in a lack of conflict over incongruence. Alternatively, perhaps early childhood teachers understand that it is in their classrooms that preschool-aged children are often exposed to requirements to be social and focused for the first time, so these traits are not as heavily considered early on in a child’s preschool experience. As a result, parents may be more concerned about social and obedience deficits in the classroom that teachers typically find developmentally normal. If this is the case, both adult actors are working appropriately to help students improve sociability, focus, and obedience, even though they view the children differently. Trained educators may be able to use typical preschool approaches like play and experimentation to help children become more sociable, focused, and obedient in ways that may seem new to parents [35]. Additionally, fearlessness is an attribute similar to confidence, which was significant in our models, so its lack of significance in social development is unexpected. It may be that fearlessness is viewed as a negative trait in small children; for example, adults would prefer that children learn respect for crosswalks rather than darting fearlessly into the street. If fearlessness is not viewed as a prosocial trait in very young children, it might be less surprising that any incongruence between parents and teachers assessing it is not associated with child development. However, confidence may be interpreted as a desirable trait that can unlock other skills. More research into this nuance could help explain what traits parents and teachers view as most important in promoting child development. Similarly, we also expected that obedience would be useful in cultivating positive relationships with caregivers, and that a similar lack of congruence on obedience might demonstrate parents being more indulgent of slow development than teachers who must manage many children from different backgrounds. The absence of significance in the divergence of parent and teacher perceptions of a child’s obedience is intriguing. We note that while these behavioral trait perceptions are not significantly associated with social and cognitive development in this study, their influence may be present in other forms of development or development in later stages of the life course.

We did find evidence that incongruence on other important traits was indeed associated with worse developmental outcomes. We found that the more teachers and parents differed in their perceptions of children being hungry for knowledge, understanding things quickly, being talkative, being confident, and being good-natured, the greater the negative impact was on the child’s social, concentration, linguistic, mathematical, and cognitive abilities, as well as knowledgeability, compared to their peers. For some traits, links between disagreement on those traits and the developmental outcomes with which they are associated seem clear: for example, incongruence in how talkative parents and teachers view a child is most associated with linguistic and knowledgeability development, which makes sense given that children are most likely to be able to express language and knowledge growth through speech. Talkativeness is a trait that can influence how parents and teachers perceive and interact with young children. In the case of our study, parents and teachers diverging on how talkative they perceive a child has negative ramifications on general knowledgeability and linguistic development compared to peers. When children are more verbally active, teachers may respond with more direct, social learning strategies to help them navigate forming new relationships [36]. Teachers may not have the same dynamic perception of quiet children, which may lead to less linguistic engagement [37]. While biases based on how outgoing a child is at school may very well be unconscious, they still have the capacity to influence teacher relationships and teaching strategies with individual students. These biases may be incongruent with how a child is viewed by their parents, leading to variance in how talkative a child is perceived and, subsequently, how effective knowledgeability and linguistic development may be. This incongruence can be lessened by parents actively involving themselves in their child’s relationship with their teacher. The more parents understand about how a teacher views their child, the more both caregivers can collaborate on establishing a joint perception of the child, their proclivities, and their relationship needs.

Similarly, incongruence in perceptions of confidence is most clearly related to social development. Caregivers can help a child cultivate confidence in social settings by instigating productive interactions from an early age [38]. Believing in a child’s ability to succeed and allowing them opportunities to showcase their work and success [39] can help increase a child’s sense of self-confidence. When parents and teachers disagree on how confident a child is, however, children miss out on such developmental opportunities. Understanding this can help educators and policymakers emphasize the importance of good communication and coming to an agreement between parents and teachers in ways that provide opportunities for social development in settings beyond the home and school.

**Figure 6:**
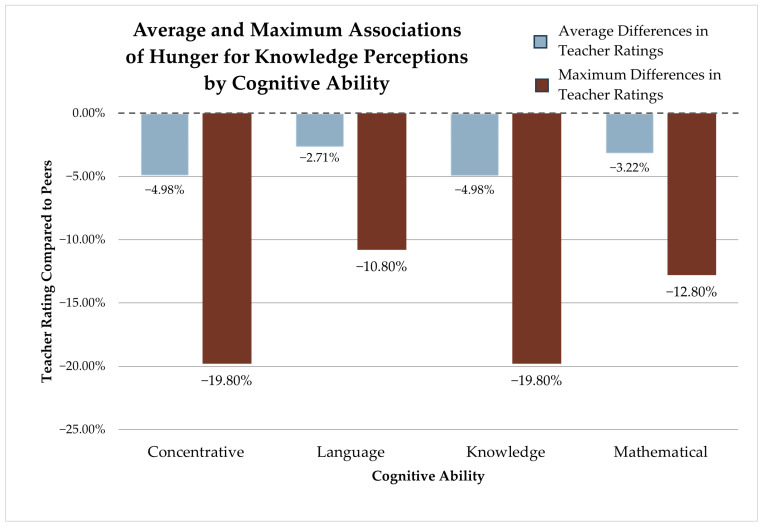
Average and maximum associations of hunger for knowledge perceptions by cognitive ability.

Being able to understand things quickly is related to different kinds of academic development but not to social development, which may reflect how academic or knowledge-based development is understood and measured. Parent–teacher perception differences of a child’s hunger for knowledge and ability to understand had some of the most negative associations with development, particularly in the areas of concentration and general knowledge abilities. If parents and teachers differ to the maximum extent on how they view a child’s hunger for knowledge, almost a 20% reduction in concentrative and general knowledge development abilities would be expected compared to their peers. See, for example, Figure 6, which shows the associations between the average difference in behavioral perception (in this case, hunger for knowledge) and four out of the five developmental abilities (blue bars on the left), along with maximum potential incongruence between parent and educator assessments of behavior (red bars on the right). Even when parents and teachers differ to an average extent (~2.5 points) on a child’s hunger for knowledge, an almost 5% lower development rating is expected for concentrative and general knowledgeability. These areas of development are two facets of growth that are particularly targeted in education. Perhaps as children try to master a new learning environment, parents’ and teachers’ views on behaviors related to topic mastery become more critical for a child to adjust to the new sphere. Children perceived as more mature or with a greater ability to handle navigating unfamiliar school settings are typically expected to do better in terms of adjustment and comprehension than others [40]. Additionally, prosocial attitudes in children, such as extraversion or outward tenacity, may lead to stronger teacher biases when rating children in cognitive development [36]. This serves to justify that parent and teacher perceptions of child behavior and ability can make a difference when it comes to initial acclimatization to preschool or daycare settings. Understanding the significance of such incongruities should spur a greater desire in both parents and teachers to communicate clearly, particularly about a child’s learning needs, interests, and aptitudes.

By contrast, incongruence on whether teachers and parents think a child is good-natured is not associated with social development, an unexpected finding, but instead is most closely associated with general knowledgeability development, where a theoretical connection is not as clear. Good-naturedness can be a notable catalyst in cultivating a positive or negative parent–child or teacher–child relationship. These relationships can direct how behavioral issues are addressed. Poor child temperament may lead to declining mental health in parents [41] or be a result of ineffectiveness of a parent to curb bad behavior because of a lack of mental health fortitude [17]. Additionally, child temperament may diminish the potential for a positive teacher–student relationship [20]. In our study, we found that differences in how parents and teachers perceive a child’s good-naturedness are negatively associated with general knowledgeability development. In a school setting, children of better temperaments may be more open-minded to learning or may be more inclined to learn new subjects when they have a positive relationship with their teacher. On the other hand, better assessments of knowledge development may only indicate that the pragmatic relationship between a child and their caregivers is more pleasant, leading teachers to rate development higher. The opposite may hinder general knowledgeability development, as was highlighted in this study. Perhaps teachers who like a child better miss subtle cues about intellectual development that parents see in more intimate settings. A greater incongruence in how parents and teachers perceive a child’s good-naturedness may lead to a child missing out on learning opportunities in one environment or the other. When parents and teachers agree on a child’s disposition, similar tactics for teaching may be employed, but a mismatch in strategies or a belief about the need for intervention can occur with greater incongruence in good-naturedness perceptions. While our findings are suggestive that gaps between parents and educators, evidenced by incongruence in their opinions about children, can be damaging to child development, a more nuanced investigation of specific types of investment and development is needed to understand these patterns.

Surprisingly, differing opinions on the neatness of a child lead to better ratings on social and cognitive developmental abilities across all five categories. This could be due to the differing priorities of learning and play in home and school environments. At home, children are exposed to family members tidying up, and they may even participate in simple household chores themselves. Cleanliness is normally reinforced at home. At school, however, children are often encouraged to use their imagination to design stories and games with a myriad of toys and other instruments, which previous research has found to help facilitate creativity and maturity through “messy play” [42]. Messy play allows children to explore curiosities, and those surveyed about messy play often cite it as beneficial for early development [43]. Both adult- and child-led activities accommodating a lack of neatness can augment a child’s social and cognitive learning experiences [44]. Given the different roles children play at home and at school, family member or student, different abilities and cleanliness practices are emphasized in each environment. While this is to be expected, a caregiver’s ability to anticipate priorities and skills emphases outside their own scope can effectively promote consistent learning opportunities across all environments of a young child’s life.

The results of this study can help improve child development outcomes in multiple settings. At home, parents should seek to establish a positive relationship with a child’s educators, as doing so has been shown to improve a teacher’s perception of a child’s behaviors and abilities [26]. Parents can also be proactive in supporting child development outside the home by equipping the child with both positive coping mechanisms and support to practice emotional regulation. Parents should be especially conscious about how their class background [25] or their devotion to their child might prevent them from understanding educators’ perspectives in ways that can block key developmental resources for their child. Establishing communication strategies to maximize involvement in a child’s learning goals and environment could enable parents to more effectively present a child’s individual needs to their teacher and facilitate a joint plan for accelerating learning opportunities. This could take the form of more frequent parent–teacher meetings, opportunities for parents to assist in a child’s class, improved technological interfaces for academic and behavioral progress to be reported, or institution-led events to increase parental trust in teachers and educational procedures [45,46].

For educators, consulting with parents to provide a safe learning atmosphere for children of different backgrounds and experiences can maximize children’s social and cognitive growth. Additionally, being aware that parents may not assess their child’s abilities well may help teachers identify which children are in need of greater individual support. Educators may learn from findings such as ours that they need to find new approaches or language to express information about child traits and development to parents who, while experts in their own children, do not have training in child development. Teacher training should also include more emphasis on teachers discussing a wider range of behavioral traits, such as those we show here are associated with development, instead of focusing parent–teacher discussions solely on traditional academic outcomes. This may be especially important in early childhood education [45,46]. Doing so can contribute to providing equal access to educational and social opportunities for all children.

For policymakers, understanding how different social environments, in this case, home and daycare or early childhood education, prioritize different traits and abilities is important for innovating effective means to support caregivers in both parenting and teaching capacities. Developing training for parents and teachers to gain a joint view of a child’s abilities, personality, and needs may help to reduce this behavior-perception incongruence that this study found to be detrimental to early social and cognitive development. Policymakers responsible for developing educational curricula should also focus on training teachers in communication skills with parents. Finally, policymakers should consider creating employment and economic structures that allow greater time for parents and teachers to meet more often to discuss child development.

As with any research on behavior and development at a social and cognitive level, this study has a couple of key limitations. The first is that due to the nature of the data and German privacy laws, controlling for daycare institution type was not possible. While the majority of children in this study attended a state-funded daycare, we could not take into account institution type or quality in our models. Similarly, information relating to the educators whose responses were used was also not available. It is also true that we used only educator ratings of the five developmental ability outcome measures. It might be useful to examine parents’ judgment of this development. Educators are more experienced and better trained in comparing students’ developmental abilities to their peers. They spend multiple hours each day with children of the same age and thus can better discern differences in development across children. Teachers are also typically trained to cultivate and assess different facets of development for a particular age group, which equips them with the breadth of knowledge and background required to gauge such competencies. While parents may be equally skilled at assessing their own child’s growth, they are less adept at making comparisons across children. Because the development measures available in the NEPS are comparisons across children, we are more confident using these teacher measures. Teachers are also typically trained to cultivate and assess different facets of development for a particular age group, which equips them with the breadth of knowledge and background required to gauge such competencies. However, it is true that without parent assessments of outcomes, we were unable to fully test this assertion. Future work using models like the ones we used here to compare not just trait assessment incongruence but teacher and parent measures of developmental skills will be an important next step. It may also be useful for researchers using the restricted-use NEPS to use institutional identifiers to confirm our assumption that there is no notable shared error among target children. Such a strategy would also allow for an in-depth examination into whether school characteristics are influential in opening or closing gaps between educators and parents or in moderating the effects of any such gaps. Additionally, we used cross-sectional data in our analysis, so examining developmental outcomes later in childhood was not included. This was a result of our interest in using teacher assessments of current students, which are cross-sectional in nature, as longitudinal research involving the child’s development prior to preschool would not include future teacher ratings of social and cognitive ability. Future work incorporating subsequent waves of data would reveal whether these patterns hold true as children grow older and more self-sufficient.

Future work may also include looking at a number of potential moderators. Gender, for example, was a significant control in this study, and evaluating how parent and teacher behavioral perceptions differ by gender for social and cognitive development may be enlightening. Previous work has shown that parents may vary parenting styles in terms of conflict management, play, and socialization based on both their own gender and that of their children [47]. Teachers may also report better relationship quality with female students [2], thus establishing the pertinence of studying gender as a moderator. Additionally, looking into how the influence of parent and teacher perceptions on development increases or decreases as a child gets older may serve to inform communication styles between parents and teachers throughout childhood and adolescence. Studies have shown that as children progress further in their schooling years, parental influence and involvement may decrease [48], which could affect the relationship between parent–teacher congruence and a child’s development in adolescence. Another consideration would be factoring in how a child’s mental health is affected by diverging parent and teacher perceptions. Parents are granted the opportunity to interact extensively with their child on an individual basis, whereas teachers often interact with a child predominantly in a group setting, which may lend greater strength to one caretaker or the other in recognizing and addressing the child’s mental health needs [49]. Given the emotional weight attached to mental wellness, disagreement between parents and teachers may yield poorer development in children experiencing mental struggles. Finally, measuring whether parent or teacher ratings have a stronger negative influence, or whether it matters which actor has a more favorable opinion of the child, may help to eliminate potential biases and raise awareness for proactive techniques to support children of different dispositions in developing early social and cognitive abilities. Parents’ rating a child more favorably may result from a desire to portray their child in a positive manner to others [33]. Parent ratings may also be higher by employing positive conflict-resolution and emotional regulation techniques with their child [21], thus leading to more favorable perceptions of behavior. Parents are a child’s first socializers, which may give weight to the possibility that their perceptions of their child have more of a stake in early development. However, given teachers’ extensive interaction with numerous preschool-age children, they may have more theoretically backed training to intervene in child behavioral deficits and foster positive change [26]. This paper solely studied the amount parents and teachers differed in their perceptions. A future paper evaluating the influence of directionality would serve to augment the insights uncovered in the present study.

The home and the daycare often serve as a child’s earliest settings for social and cognitive development. With parents and educators typically being a child’s first long-term caregivers, understanding a child’s full personality, needs, and growth is important to support an upward trajectory of development. While different social or intellectual abilities are prioritized differently in each setting, parents and teachers carry a large responsibility to reinforce positive behaviors and aid children in learning new comprehension and interaction abilities. This responsibility should be shared by both parents and teachers to maximize the success young children can have in cultivating early social and cognitive growth.

## Figures and Tables

**Table 1:** Descriptive statistics of social and cognitive development measures.

Developmental Ability	Mean	Standard Deviation
Social	3.201	0.911
Concentration	3.112	0.994
Language	3.124	1.020
General Knowledgeability	3.190	0.901
Math	3.114	0.869
N = 2968		

Source: NEPS SC 2, Wave 2, 2011/2012.

**Table 2:** Descriptive statistics of differences in parent and teacher perceptions of the child’s behavior.

Behavioral Trait (Prosocial)	Mean	Standard Deviation
Talkative	2.473	2.051
Neat	2.817	1.928
Good-natured	2.994	2.013
Hungry for Knowledge	2.513	2.078
Confident	2.817	2.084
Sociable	2.371	1.858
Focused	2.755	1.972
Obedient	2.774	1.801
Understanding	2.705	2.157
Fearless	2.981	2.056
N = 2968		

Source: NEPS SC 2, Wave 2, 2011/2012.

**Table 3:** Descriptive statistics of control variables.

Variable	Range	Mean/Proportion (%)	Standard Deviation
Household Size (Aggregated)	1–6	3.958	0.964
Monthly HH Income (Agg.)	0–6	3.332	1.257
Assessment of HH Economic Situation	1–5	1 “Very Bad” (2.83), 2 “Rather Bad” (7.99), 3 “Partly Good” (31.20), 4 “Rather Good” (41.81), 5 “Very Good” (16.17)	Not Applicable (NA)
Primary Parent Employment Status	1–4	1 “Full-time” (19.07), 2 “Part-time” (39.56), 3 “Side-job” (7.85), 4 “Unemployed” (33.52)	NA
Primary Parent Married, Lives with Spouse	0–1	0 “No” (21.53), 1 “Yes” (78.47)	NA
Partner Child’s Bio Parent	0–1	0 “No” (12.63), 1 “Yes” (87.37)	NA
Mother’s Age at Child’s Birth	14–64	30.911	5.795
Primary Parent Education Attainment	9–18	13.613	2.267
Partner Education Attainment	9–18	13.413	2.362
Children Face Pressure to Perform at School	1–4	1 “Does Not Apply” (9.40), 2 “Does Rather Not Apply” (27.19), 3 “Does Rather Apply” (32.95), 4 “Does Apply” (30.46)	NA
Weaker Performance Means Less Support	1–4	1 “Does Not Apply” (21.73), 2 “Does Rather Not Apply” (36.69), 3 “Does Rather Apply” (23.69), 4 “Does Apply” (17.89)	NA
Demands too High in Elementary School	1–4	1 “Does Not Apply” (11.19), 2 “Does Rather Not Apply” (24.53), 3 “Does Rather Apply” (29.18), 4 “Does Apply” (35.11)	NA
Losing Fun in Learning in Elementary School	1–4	1 “Does Not Apply” (37.33), 2 “Does Rather Not Apply” (39.32), 3 “Does Rather Apply” (13.24), 4 “Does Apply” (10.71)	NA
Importance of Preparing Child for School	1–4	1 “Not Important At All” (1.82), 2 “Rather Not Important” (11.05), 3 “Rather Important” (24.29), 4 “Very Important” (62.84)	NA
Child Birthweight	330–6000	3339.230	612.705
Child Gender	0–1	0 “Girl” (49.36), 1 “Boy” (50.64)	NA
N = 2968			

Source: NEPS SC 2, Waves 1 & 2, 2010/2012.

**Table 4:** Association between developmental abilities and parent–educator behavioral perception incongruence.

Behavior Trait	Social Coefficient (Std. Coeff.)	Concentration Coefficient (Std. Coeff.)	Language Coefficient (Std. Coeff.)	Knowledge Coefficient (Std. Coeff.)	Mathematics Coefficient (Std. Coeff.)
Talkative	0.010 (0.023)	0.012 (0.025)	−0.035 ** (−0.070) **	−0.028 * (−0.065) *	−0.020 (−0.048)
Neat	0.030 ** (0.063) **	0.030 *** (0.058) ***	0.044 *** (0.083) ***	0.024 * (0.050) *	0.026 * (0.057) *
Good-Natured	0.017 (0.038)	0.000 (−0.001)	−0.008 (−0.016)	−0.026 ** (−0.058) **	−0.010 (−0.024)
Hungry for Knowledge	−0.073 *** (−0.162) ***	−0.099 *** (−0.204) ***	−0.054 *** (−0.109) ***	−0.099 *** (−0.225) ***	−0.064 *** (−0.151) ***
Confident	−0.024 * (−0.055) *	−0.013 (−0.028)	−0.009 (−0.020)	−0.016 (−0.037)	−0.005 (−0.013)
Sociable	−0.010 (−0.020)	−0.002 (−0.003)	−0.022 (−0.040)	−0.006 (−0.012)	0.005 (0.011)
Focused	−0.004 (−0.008)	−0.004 (−0.008)	0.013 (0.024)	−0.001 (−0.002)	0.003 (0.006)
Obedient	0.007 (0.013)	−0.010 (−0.018)	−0.019 (−0.034)	−0.007 (−0.014)	−0.001 (−0.001)
Understands Quickly	−0.006 (−0.014)	−0.092 *** (−0.200) ***	−0.082 *** (−0.172) ***	−0.05 *** (−0.121) ***	−0.080 *** (−0.199) ***
Fearless	0.000 (−0.009)	−0.008 (0.016)	0.016 (0.033)	0.014 (0.033)	0.021 (0.049)
N = 2968					

Source: NEPS SC 2, Waves 1 & 2, 2011/2012. * *p* < 0.05, ** *p* < 0.01, *** *p* < 0.001. These models controlled for household size, monthly household income, assessment of household economic situation, primary parent employment status, primary parent marital status, primary parent’s partner’s relationship to child, primary parent migrant status, mother’s age at birth of child, primary parent educational attainment, primary parent’s partner educational attainment, primary parent educational attitudes, child birthweight, and child sex.

## Data Availability

No new data were created or analyzed in this study. Data may be obtained from the Leibniz Institute of Educational Trajectories by application through doi:10.5157/NEPS:SC2:1.0.0. This paper uses data from the National Educational Panel Study (NEPS): Starting Cohort 2–Kindergarten, doi:10.5157/NEPS:SC2:1.0.0. We accessed the data for this project starting 24 September 2024. From 2008 to 2013, NEPS data were collected as part of the Framework Programme for the Promotion of Empirical Educational Research funded by the German Federal Ministry of Education and Research (BMBF). As of 2014, the NEPS survey is carried out by the Leibniz Institute for Educational Trajectories (LIfBi) at the University of Bamberg in cooperation with a nationwide network.

## Abbreviations

The following abbreviations are used in this manuscript:

NEPS	National Educational Panel Study, Starting Cohort 2
OLS	Ordinary Least Squares regression
LIfBi	Leibniz Institute of Educational Trajectories
KMK	Standing Conference of the Ministers of Education and Cultural Affairs
SC 2	National Educational Panel Study, Starting Cohort 2
SE	Standard Error

## References

[1] Howes C., James J. (2002). Children’s social development within the socialization context of childcare and early childhood education. Blackwell Handbook of Childhood Social Development.

[2] Zhu Y., Li X., Jiao D., Tanaka E., Tomisaki E., Watanabe T., Sawada Y., Zhu Z., Ajmal A., Matsumoto M. (2021). Development of social skills in kindergarten: A latent class growth modeling approach. Children.

[3] Brazzelli E., Grazzani I., Pepe A. (2021). Promoting prosocial behavior in toddlerhood: A conversation-based intervention at nursery. J. Exp. Child Psychol..

[4] Maggi S., Irwin L.J., Siddiqi A., Hertzman C. (2010). The social determinants of early child development: An overview. J. Paediatr. Child Health.

[5] Goswami U., Bryant P. (2007). Children’s Cognitive Development and Learning.

[6] Blair C., McKinnon R.D., Daneri M.P. (2018). Effect of the tools of the mind kindergarten program on children’s social and emotional development. Early Child. Res. Q..

[7] Feinstein L. (2003). Inequality in the early cognitive development of British children in the 1970 cohort. Economica.

[8] Benner A.D., Boyle A.E., Sadler S. (2016). Parental involvement and adolescents’ educational success: The roles of prior achievement and socioeconomic status. J. Youth Adolesc..

[9] Attig M., Weinert S. (2020). What impacts early language skills? Effects of social disparities and different process characteristics of the home learning environment in the first 2 years. Front. Psychol..

[10] Dang H.H., Rogers F.H. (2016). The decision to invest in child quality over quantity: Household size and household investment in education in Vietnam. World Bank Econ. Rev..

[11] Santiago R.T., Garbacz S.A., Beattie T., Moore C.L. (2016). Parent-teacher relationships in elementary school: An examination of parent-teacher trust. Psychol. Sch..

[12] Garbacz S.A., Sheridan S.M., Koziol N.A., Kwon K., Holmes S.R. (2015). Congruence in parent–teacher communication: Implications for the efficacy of CBC for students with behavioral concerns. Sch. Psychol. Rev..

[13] Baumrind D. (1978). Parental disciplinary patterns and social competence in children. Youth Soc..

[14] Baumrind D. (1975). The contributions of the family to the development of competence in children. Schizophr. Bull..

[15] Rucinski C.L., Brown J.L., Downer J.T. (2018). Teacher–child relationships, classroom climate, and children’s social-emotional and academic development. J. Educ. Psychol..

[16] Herrera M., Little E. (2005). Behaviour Problems across Home and Kindergarten in an Australian Sample. Aust. J. Educ. Dev. Psychol..

[17] Phares V., Compas B.E., Howell D.C. (1989). Perspectives on child behavior problems: Comparisons of children’s self-reports with parent and teacher reports. Psychol. Assess. J. Consult. Clin. Psychol..

[18] Kohl G.O., Lengua L.J., McMahon R.J. (2000). Parent involvement in school conceptualizing multiple dimensions and their relations with family and demographic risk factors. J. Sch. Psychol..

[19] Miller L.S., Koplewicz H.S., Klein R.G. (1997). Teacher ratings of hyperactivity, inattention, and conduct problems in preschoolers. J. Abnorm. Child Psychol..

[20] Zhang X., Sun J. (2011). The reciprocal relations between teachers’ perceptions of children’s behavior problems and teacher–child relationships in the first preschool year. J. Genet. Psychol..

[21] Spinrad T.L., Gal D.E. (2018). Fostering prosocial behavior and empathy in young children. Curr. Opin. Psychol..

[22] Huang W., Weinert S., Wareham H., Law J., Attig M., von Maurice J., Roßbach H. (2022). The emergence of 5-year-olds’ behavioral difficulties: Analyzing risk and protective pathways in the United Kingdom and Germany. Front. Psychol..

[23] Albanese A.M., Russo G.R., Geller P.A. (2019). The role of parental self-efficacy in parent and child well-being: A systematic review of associated outcomes. Child Care Health Dev..

[24] Jeon L., Buettner C.K., Snyder A.R. (2014). Pathways from teacher depression and child-care quality to child behavioral problems. J. Consult. Clin. Psychol..

[25] Lareau A. (2018). Unequal Childhoods: Class, Race, and Family Life.

[26] Minke K.M., Sheridan S.M., Kim E.M., Ryoo J.H., Koziol N.A. (2014). Congruence in parent-teacher relationships: The role of shared perceptions. Elem. Sch. J..

[27] Peisner-Feinberg E.S., Burchinal M.R., Clifford R.M., Culkin M.L., Howes C., Kagan S.L., Yazejian N. (2001). The relation of preschool child-care quality to children’s cognitive and social developmental trajectories through second grade. Child Dev..

[28] Phillips D., McCartney K., Scarr S. (1987). Child-care quality and children’s social development. Dev. Psychol..

[29] Hughes J.N., Gleason K.A., Zhang D. (2005). Relationship influences on teachers’ perceptions of academic competence in academically at-risk minority and majority first grade students. J. School Psychol..

[30] Blossfeld H., Von Maurice J. (2011). 2 Education as a lifelong process: The German National Educational Panel Study (NEPS) [Special Issue]. Z. Erzieh..

[31] Ghosh S., Kleine L. (2025). School entry-age effect on student’s affective–motivational attitudes in German elementary schools. Early Child. Educ. J..

[32] Mikus K., Tieben N., Schober P.S. (2021). Concerted cultivation in early childhood and social inequalities in cognitive skills: Evidence from a German panel study. Res. Soc. Stratif. Mobil..

[33] Kårstad S.B., Kvello Ø., Wichstrøm L., Berg-Nielsen T.S. (2014). What do parents know about their children’s comprehension of emotions? Accuracy of parental estimates in a community sample of pre-schoolers. Child Care Health Dev..

[34] Ready D.D., Wright D.L. (2011). Accuracy and inaccuracy in teachers’ perceptions of young children’s cognitive abilities: The role of child background and classroom context. Am. Educ. Res. J..

[35] Pramling Samuelsson I., Björklund C. (2023). The relation of play and learning empirically studied and conceptualised. Int. J. Early Years Educ..

[36] Coplan R.J., Hughes K., Bosacki S., Rose-Krasnor L. (2011). Is silence golden? Elementary school teachers’ strategies and beliefs regarding hypothetical shy/quiet and exuberant/talkative children. J. Educ. Psychol..

[37] McCroskey J.C., Richmond V.P. (1991). Quiet Children and the Classroom Teacher.

[38] Miller S.R., Coll E. (2007). From social withdrawal to social confidence: Evidence for possible pathways. Curr. Psychol..

[39] Sarkowi S., Widat F., Wadifah N.I., Rohmatika D. (2023). Increasing children’s self-confidence through parenting: Management perspective. J. Obs. J. Pendidik. Anak Usia Dini.

[40] Jewsuwan R., Luster T., Kostelnik M. (1993). The relation between parents’ perceptions of temperament and children’s adjustment to preschool. Early Child. Res. Q..

[41] Putnam S.P., Sanson A.V., Rothbart M.K., Bornstein M.H. (2002). Child temperament and parenting. Handbook of Parenting.

[42] Yin L.C., Zakaria A.R., Baharun H., Hutagalung F., Sulaiman A.M. (2015). Messy play: Creativity and imagination amount preschool children. Interdisciplinary Behavior and Social Sciences.

[43] Casey L., Prendiville S. (2020). Enhancing and enriching children’s learning and development through messy play. Routledge International Handbook of Play, Therapeutic Play and Play Therapy.

[44] Gascoyne S. (2018). Messy Play in the Early Years: Supporting Learning Through Material Engagements.

[45] Levin O. (2024). Emotional, behavioural, and conceptual dimensions of teacher-parent simulations. Teach. Teach..

[46] Smith T.E., Sheridan S.M. (2019). The effects of teacher training on teachers’ family-engagement practices, attitudes, and knowledge: A meta-analysis. J. Educ. Psychol. Consult..

[47] Morawska A. (2020). The effects of gendered parenting on child development outcomes: A systematic review. Clin. Child Fam. Psychol. Rev..

[48] Murray E., McFarland-Piazza L., Harrison L.J. (2015). Changing patterns of parent–teacher communication and parent involvement from preschool to school. Early Child Dev. Care.

[49] Carneiro A., Soares I., Rescorla L., Dias P. (2021). Meta-analysis on parent–teacher agreement on preschoolers’ emotional and behavioural problems. Child Psychiatry Hum. Dev..

